# The child dental care reform in Israel - age-related patterns of uptake: 2011 to 2022

**DOI:** 10.1186/s13584-025-00714-3

**Published:** 2025-08-25

**Authors:** Dan Dekel, Hazav Dadosh, Hagit Domb Herman, David Yellon, Shlomo Paul Zusman, Lena Natapov

**Affiliations:** Division of Dental Health, Ministry of Health, Jerusalem, Israel

**Keywords:** Universal health coverage, Child dental care reform, Public health policy, Age-specific dental care utilization, Prevention

## Abstract

**Background:**

The Child Dental Care Reform introduced in Israel in 2010 aimed to provide universal dental coverage for children, addressing high caries morbidity and inequalities in access to care. The reform initially covered ages 0–8 and expanded to include all children up to age 18 by 2019. This study examines age-related patterns of dental service utilization during the first decade of its implementation.

**Methods:**

This retrospective study analyzed anonymized dental service data from 2011 to 2022, submitted by the four Health Maintenance Organizations to the Israeli Ministry of Health. The data included the number of children treated, categorized by age group, and the types of treatments provided.

**Results:**

Service utilization showed distinct age-related patterns, with rates peaking at age 8 (48%) and gradually declining through adolescence (*p* < 0.001). Restorative care consistently outnumbered preventive care across all age groups (*p* < 0.001), with children aged 3–5 receiving the most restorative procedures per child. Preventive treatments increased with age, from 1.0 per patient in young children to 1.5 in teenagers, transitioning from mainly dental examinations in younger children to hygienist visits in adolescents. Restorative treatments included dental restorations (peaking at 50% at ages 8–9), extractions (25% at ages 10–11), and pulp treatments (25% at ages 6–8). Emergency dental visits were most common in infants and increased by 83% over the course of a decade (*p* < 0.001). General anesthesia utilization increased significantly in the younger age groups, with the 4–5 age group showing the most dramatic increase (2.39-fold increase, *p* < 0.001).

**Conclusion:**

This study highlights distinct age-related patterns in dental service utilization among children in Israel, emphasizing the need for targeted prevention strategies and policy reforms to address current challenges disparities, including the increasing rate of treatment under general anesthesia. Preventive interventions, such as community water fluoridation and early childhood programs, alongside improved access to specialized dental care, are essential for fostering better long-term oral health outcomes. Integrating quality indicators will facilitate better incorporation of dental services into the national health system, ensuring comprehensive and equitable oral care.

## Background

Oral health is essential for children’s well-being, impacting various aspects of their lives, from physical comfort to self-esteem and general health. Dental caries is the most prevalent noncommunicable disease globally, affecting almost half of the world’s population and accounting for 5–10% of healthcare budgets in industrialized countries [1]. Poor oral health is related to chronic diseases and can profoundly affect general health across all age groups [2]. Children and adolescents are particularly vulnerable, with oral diseases being among the main reasons for pediatric hospitalization in some high-income countries, and sociodemographic factors remain key predictors for hospital admission of children with dental caries [3].

Early childhood caries (ECC) is a public health problem affecting children worldwide. It is defined as one or more decayed, missing, or filled tooth surfaces (due to caries) in any primary tooth in a child younger than six years. Severe ECC can lead to extensive tooth destruction, requiring dental treatment under general anesthesia (GA) and hospitalization. This condition affects children’s quality of life by disrupting daily activities, school attendance and learning ability [4], highlighting the essential role of oral health in children’s overall well-being [5].

Dental caries among children in Israel is a significant public health challenge. Previous studies described high caries morbidity and a high prevalence of untreated disease, particularly among socioeconomically disadvantaged groups [6]. A recent study revealed that dental health problems can impact children’s future social mobility by affecting their school attendance and academic performance [7].

The Child Dental Care Reform (CDCR) was introduced in Israel in 2010 under the National Health Insurance Law (NHIL), aiming to reduce inequalities and provide universal dental care coverage for children. The reform was implemented gradually, initially covering children aged 0–8 years in 2011 and gradually expanded to cover all children under the age of 18 by 2019. The basket of services includes a range of preventive and restorative treatments, such as routine checkups, fluoride applications, scaling, restorations and extractions. Dental treatment is offered free of charge or with minimal copayment through the four Health Maintenance Organizations (HMOs) at dental clinics across the country. The reform aligns with the 2023 World Health Organization’s (WHO) Global Strategy and Action Plan on Oral Health, which emphasizes the integration of tailored, age-appropriate oral health care into relevant national health programs [8].

Previous research has shown that although children’s dental visits have increased following the implementation of the reform, a significant portion of children still do not utilize the universally covered oral health services [9]. A lack of parental awareness of the importance of dental care at an early age, in addition to fear or anxiety, cultural beliefs, communication barriers or difficulty accessing dental services may lower the frequency of visits to the dental clinic by young children [10].

Since caries is largely preventable, early intervention is important as delayed care often results in more complex and prolonged treatments. A study analyzing treatments performed under general anesthesia in children aged 3–5 years reported that while younger children primarily received basic restorative treatments, the complexity of needed interventions increased substantially with age [11].

Understanding the patterns of oral care utilization among children of different age groups is important for evaluating the effectiveness of the reform and for planning targeted interventions to further improve the universally covered dental services. This study aims to examine age-specific dental healthcare utilization patterns in Israel from 2011 to 2022 and to assess the uptake and treatments provided under the dental care reform across different age groups.

## Methods

This retrospective study analyzed data submitted annually by the four HMOs regarding the utilization of the dental reform between 2011 and 2022, as previously described [9]. The reports included data on the number of children treated by age group, as well as the type and number of treatments provided, containing only anonymized information with no personal details that may identify the patients.

For each age group, data were analyzed only for the years following their inclusion in the reform. The data covered treatments provided within the basket of dental services for children, which included preventive treatments (examination & treatment plan, hygienist appointment, topical fluoride application, oral hygiene instructions and pit and fissure sealants) and restorative treatments (dental restorations, pulp treatments, dental posts, pediatric dental crowns, extraction, space maintainers, and emergency care).

The reports also included data on dental treatments performed under general anesthesia (included in the basket for children under the age of 5).

To examine treatment profiles, treatments were categorized by codes and grouped into preventive and restorative care.

### Ethical approval

The study was approved by the Ministry of Health Ethics Committee (MOH 119–2023).

## Results

The results are presented in the following order: overall utilization patterns, further analyzed by care type and specific treatment categories.

### Demographics and utilization rates

The number of eligible children increased from 1,546,857 in 2011 to 3,178,238 in 2022, reflecting the progressive inclusion of additional age groups in the NHIL reform [9].

**Fig. 1:**
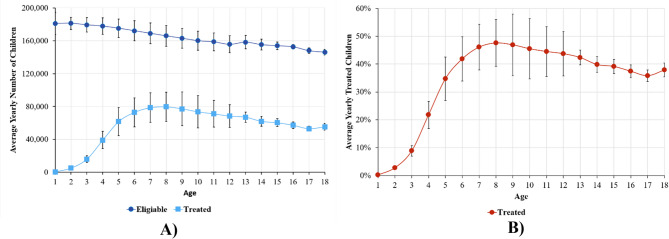
Age-specific patterns of dental care utilization, 2011–2022. **(A)** Average annual number of eligible and treated children by age, calculated from the year of inclusion in the reform. **(B)** Average utilization rates by age, calculated as the percentage of eligible children who received treatment. The error bars indicate the standard deviation

Figure 1 illustrates the age-specific patterns of dental care eligibility and average yearly utilization rates. The data reveal age-dependent trends in dental care utilization. Despite representing the largest fraction of insured children (Fig. 1A), those under 3 years of age had the lowest utilization rate, ranging from 0.2 to 10% (Fig. 1B). Utilization rates gradually increase with age, with a peak of 48% at age 8. Between the ages of 7 and 11, the utilization rate exceeds 45%. A gradual decline is observed in the middle-to-late teenage years, with 38% uptake at age 18. (*p* < 0.001).

### Preventive and restorative care

**Fig. 2:**
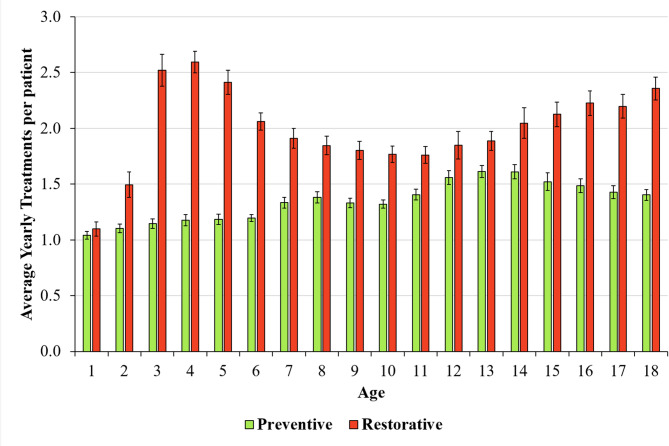
Average number of preventive and restorative treatments per patient 2013–2022

When the average number of preventive and restorative treatments per patient was compared, restorative treatments outnumbered preventive treatments across all age groups (Fig. 2).

Restorative treatments reach their highest levels in early childhood (ages 3–5), followed by a gradual decrease to approximately 1.8 treatments per patient in older children (ages 7–13), with a slight increase in late adolescence. In contrast, preventive treatments show a steady and gradual increase with age, starting at approximately 1.0 treatment per patient for the youngest children and increasing to approximately 1.5 for teenagers. (*p* < 0.001).

**Fig. 3:**
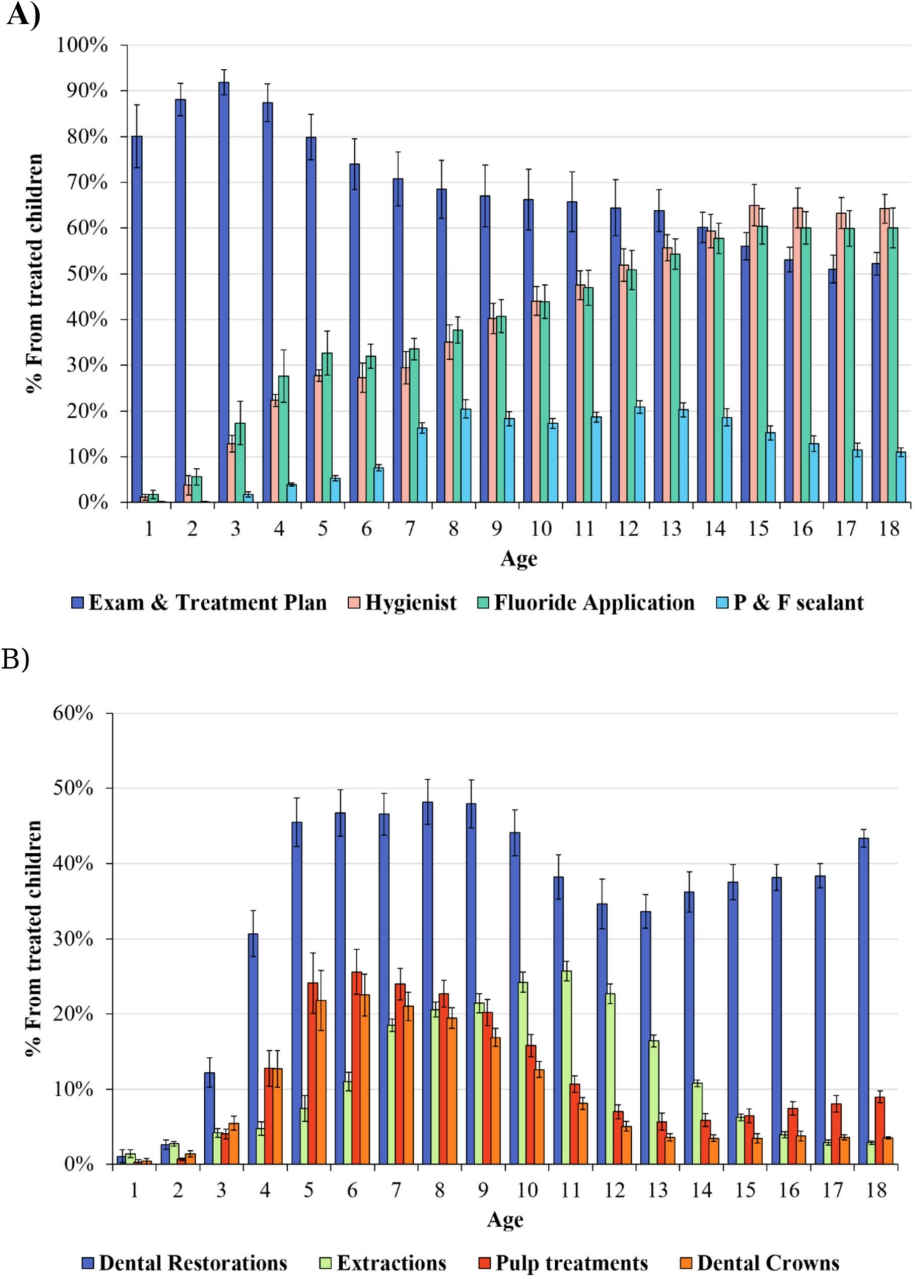
Utilization pattern of the basket of services offered by the CDCR 2013–2022. **(A)** Preventive care utilization pattern by age. **(B)** Restorative treatment utilization pattern by age

To examine the most common treatments in different age groups, the average percentage of patients who received each treatment code was calculated for 2013–2022 (Fig. 3).

For preventive care (Fig. 3A), dental exams and treatment plans had the highest utilization rates among treated children younger than 14 years of age, peaking at over 90% in children aged 2–4 years and gradually declining to approximately 60% for 18-year-olds. For older teens, the most common preventive treatments were hygienist appointments and fluoride applications, with a 60–65% utilization rate in treated children aged 15–18 years.

The highest utilization rate of pit and fissure sealants was observed in children aged 12–13 years (22–24%).

Regarding restorative treatments (Fig. 3B), dental restorations were the most frequently performed treatment, with utilization increasing from approximately 10% at age 3 to almost 50% at ages 8–9 and exceeding 35% after the age of 14.

Extractions were relatively uncommon in very young children, but utilization increased with age, peaking at approximately 25% around ages 10–11. After that, extraction rates gradually decreased.

Pulp treatments were found to be very rare in early childhood (ages 1–3), but their incidence increased to approximately 25% in children aged 6–8 years and gradually declined in older adolescents. The utilization of dental crowns followed the pattern observed in pulp treatments but with lower overall percentages.

**Fig. 4:**
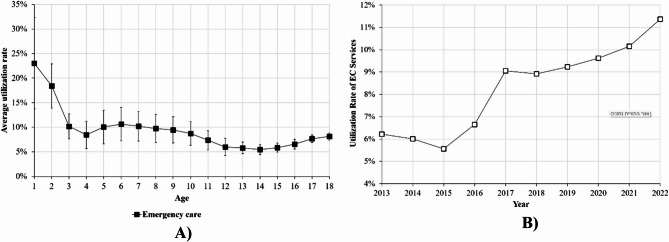
Emergency dental treatment 2013–2022. **(A)** Average utilization pattern divided by age **(B)** Utilization rate among all children) aged 0–18 years)

Figure 4A illustrates the age-related pattern in emergency dental treatment, with the highest utilization rates occurring in the first year of life. After a sharp decline by age 3, the rates stabilize approximately 5–10% through childhood, followed by a slight increase in late adolescence.

With respect to the overall changes in the rate of emergency care utilization over the years (Fig. 4B), there was a slight decline from 2013 to 2015, followed by a steady increase, with a rise to 9% in 2017 and a further rise to 11% in 2022. This represented an overall increase of 83% in emergency care utilization over the decade. (*p* < 0.001).

**Fig. 5:**
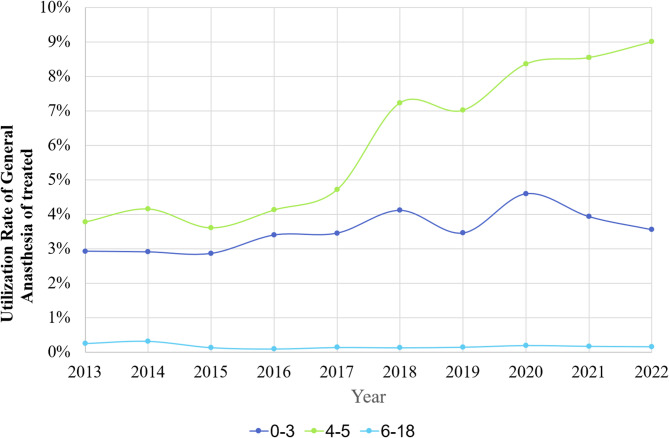
Utilization rates of general anesthesia in children by age groups, 0–18 years, in Israel from 2013 to 2022

Figure 5 illustrates the utilization rate of GA for dental treatment. The 0–3 age group demonstrated a 22% increase from 2.9% in 2013 to 3.55% in 2022. The 4–5 age group showed the most notable increase from 3.8% in 2013 to 9.1% in 2022, a 2.39-fold increase. (*p* < 0.001).

The 6–18 age group maintained the lowest rate, between 0.1% and 0.3%.

## Discussion

Addressing oral health inequalities and improving access to dental care are global public health priorities. The WHO Global Strategy and Action Plan on Oral Health calls for public policies that prioritize universal oral health coverage and promote initiatives to empower individuals and advance oral health equity [8].

Israel’s implementation of the dental care reform, which started in 2010, demonstrated such an approach, addressing preexisting challenges, including high caries morbidity and high out-of-pocket costs of dental treatment. The effects of the reform are evident and include an increase in both the number of eligible and treated children over the past decade [9]. From 2011 to 2022, the number of eligible children more than doubled, from 1,546,857 to 3,178,238, and the uptake of services increased from 8% in 2011 to 33% in 2022.

Israel’s birth rate is nearly double the OECD average (3.0 vs. 1.58) [12] and 27.8% of the population is under the age of 15, which is significantly higher than the OECD average of 17.3% [13]. With a total population of approximately 9.6 million at the end of 2022, 35% of the Israeli population was under the age of 18 and therefore was covered by the reform.

### Utilization rates

Our study results reveal distinct patterns of dental service utilization across different age groups. Children under the age of 3, despite being the largest fraction of insured children, have the lowest dental service utilization rate.

Several factors may contribute to this, such as lack of parental awareness of the importance of early dental visits and the belief that primary teeth do not require care [14]. Another factor might be that treating young children may pose a challenge due to their limited ability to cooperate, which can create anxiety for both parents and children. The treatment of young children often requires specialist pediatric dentists. However, the limited availability of these specialists could create additional barriers in accessing appropriate care.

The highest utilization rates (close to 50%) were observed in 7–10-year-old children. These high rates might be due to several factors. First, this age group experiences visible changes in the dentition, which may increase parental awareness and prompt more dental visits. Additionally, children in this age group generally demonstrate better cooperation during dental procedures compared to younger children, as they can better understand explanations and instructions, and manage anxiety. This is supported by a study by Katsouda among 4-12-year-olds, which found that older children exhibited significantly more positive behavior during dental treatment [10].

The School Dental Service (SDS), which was implemented in kindergartens and schools in Israel and includes oral health education and annual dental screening tests, may also significantly contribute to the high utilization rates observed in this study. This service can help identify treatment needs, increase awareness and motivate parents to seek professional dental care for their children [16]. Studies have demonstrated its effectiveness, as evidenced by a report from the Mayers-JDC-Brookdale Institute, which identified the SDS as one of the key factors in encouraging individuals to seek dental care [15]. Additionally, research from southern Israel has shown that children who participate in supervised toothbrushing programs have a greater number of treated teeth compared to their non-participating counterparts [16]. Similarly, UK research confirmed that school-based oral health programs increased the number of dental clinic visits [17]. These findings may help explain the higher utilization rates observed in primary school children aged 7–10 years.

### Type and number of treatments per child

Differences in the average number of treatments per child were observed across age groups. Restorative treatments were significantly more common than preventive treatments, especially in the 3–5 age group, who received, on average, more than 2.5 treatments per child. A possible explanation is that this could reflect the high treatment needs of young children suffering from ECC, who often require multiple procedures, whereas preventive care typically involves only one or two procedures, even for those with high treatment needs. The decrease in restorative treatments after the age of 5 years suggests that early interventions may have a positive impact on children’s oral health. The slight increase in treatments observed at ages 15–18 may reflect new needs, which are related to the increased caries risk due to lifestyle changes at these ages, including possible orthodontic treatment, which may require additional interventions.

With respect to preventive treatments, dental examinations predominate across most age groups, with scaling procedures surpassing them during adolescence. This finding suggests that teenagers visit dental hygienists more frequently than they visit dentists. From a public health perspective, this trend presents an opportunity. Dental hygiene visits play an important role in maintaining oral health through preventive care and oral health education.

In this study, a consistently high percentage of children received restorative treatments, particularly among young age groups. The rates of extractions and pulp treatments were very high, exceeding 20% of treated patients between the ages of 5–10 years for pulp treatments and 8–12 years for extractions, suggesting that caries morbidity in childhood and early adolescence in Israel is still high. This aligns with a 2016 study showing that 61.7% of 6-year-old children suffer from dental decay, whereas only 38.3% are caries-free [9].

Emergency dental treatment rates were found to be higher in very young children. This might be connected to the overall low utilization rate of dental services at these ages. It can be assumed that young children visit the dental clinic mainly in cases of symptoms such as pain or swelling. Additionally, an increasing percentage of young children under the age of 5 years have been treated under general anesthesia. These findings may indicate a more severe burden of dental disease, especially among young children, due to gaps in preventive care and reduced exposure to optimal fluoride levels after cessation of water fluoridation in Israel in 2014. Children born after 2014 were not exposed to the protective effect of fluoridated water and may have experienced increased caries prevalence and severity. This is supported by a recent study conducted in Canada, which demonstrated an increase in the percentage of children treated under GA after cessation of water fluoridation [18]. Another possible reason for the high rates of emergency care and dental treatment under GA may be the shortage of pediatric dental specialists who are trained to treat very young children, resulting in long waiting times for treatment. However, data on waiting times were not available in this study, and further research is needed to better understand this trend.

Community-based prevention programs play a critical role in the early prevention of dental morbidity, particularly for young children who are most vulnerable to severe dental disease requiring treatment under GA. Oral health programs such as fluoride varnish applications and supervised tooth brushing in community-based settings are essential for early intervention and prevention of dental caries in young children. Developed by the Ministry of Health (MOH), these programs in Mother and Child centers (“Tipat Halav”) and kindergartens bring together public health nurses, dental professionals, and dental hygienists to provide routine checkups, parental consultations, caries risk assessments, oral health education and preventive care, including fluoride varnish applications [19].

According to the American Dental Association, implementing evidence-based, standardized quality measures is crucial for evaluating the effectiveness of care, improving transparency, and reducing variability in care quality [20]. The introduction of quality indicators for dental care in Israel can help policymakers and HMOs monitor and enhance oral health outcomes. To support these efforts, and to evaluate community-based oral health programs and trends in children’s oral health over time, a national epidemiological database should be established, in line with WHO recommendations [21]. These tools would also provide an assessment of the effectiveness of the reform and highlight specific age groups or areas of care that require targeted interventions.

The current study has several limitations. The data submitted by the HMOs to the Ministry of Health included the number of children treated and the types of treatments provided, but did not contain information on gender, socioeconomic status, or population group. Previous studies have shown that children from lower socioeconomic backgrounds tend to seek routine dental care less frequently, even when services are publicly funded [14]. In addition, although the data were based on official HMO reports that are regularly used by the MOH, occasional reporting errors cannot be entirely ruled out.

To further evaluate the reform’s impact on oral health and to better understand barriers and inequalities in access, future research should address these issues.

## Conclusions

The findings of this study reveal significant variations in dental care utilization. Differences in the use of preventive and restorative dental care among children of different age groups highlight the importance of continued efforts by HMOs and the MOH to refine their strategies and better support children’s oral health. In addition, the MOH, HMOs, and other relevant stakeholders should play an active role in raising public awareness and encouraging greater use of services, particularly among populations with lower utilization.

Primary prevention interventions should be further developed and expanded, along with the renovation and expansion of community-based oral health programs, such as water fluoridation and oral health promotion initiatives in various community and health settings, such as “Tipat Halav” and nurseries.

Measures to ensure the availability of dental treatment, particularly by specialists, should be prioritized and planned to meet the specific needs of each age group. Ensuring an adequate number of dental specialists is crucial for providing appropriate care and preventing the need for more complex treatments later, especially for younger children. Additionally, implementing quality indicators for oral health care will help accelerate the integration of dental services into the broader range of medical services covered under national health insurance.

## Data Availability

The datasets analyzed during the current study are not publicly available due to regulatory reasons but can be obtained from the corresponding author upon reasonable request.

## Abbreviations

ECC: Early Childhood Caries
GA: General Anesthesia
CDCR: Child Dental Care Reform
NHIL: National Health Insurance Law
HMO: Health Maintenance Organization
WHO: World Health Organization
OECD: Organisation for Economic Co-operation and Development
SDS: School Dental Service
MOH: Ministry of Health
